# Application of interspecific Somatic Cell Nuclear Transfer (iSCNT) in sturgeons and an unexpectedly produced gynogenetic sterlet with homozygous quadruple haploid

**DOI:** 10.1038/s41598-018-24376-1

**Published:** 2018-04-16

**Authors:** Effrosyni Fatira, Miloš Havelka, Catherine Labbé, Alexandra Depincé, Viktoriia Iegorova, Martin Pšenička, Taiju Saito

**Affiliations:** 1 Faculty of Fisheries and Protection of Waters, South Bohemian Research Center of Aquaculture and Biodiversity of Hydrocenoses, University of South Bohemia in Ceske Budejovice, Zátiší 728/II, 389 25 Vodňany, Czech Republic; 2 Faculty and Graduate School of Fisheries Sciences, Hokkaido University, 3-1-1 Minato, Hakodate, Hokkaido, 041-8611 Japan; 3 INRA, Fish Physiology and Genomics department, Campus de Beaulieu, F-35000 Rennes, France; 4 Nishiura Station, South Ehime Fisheries Research Center, Ehime University, Uchidomari, Ainan, Ehime 798-4206 Japan

**Keywords:** Cloning, Embryology

## Abstract

Somatic cell nuclear transfer (SCNT) is a very promising cloning technique for reconstruction of endangered animals. The aim of the present research is to implement the interspecific SCNT (iSCNT) technique to sturgeon; one fish family bearing some of the most critically endangered species. We transplanted single cells enzymatically isolated from a dissociated fin-fragment of the Russian sturgeon (*Acipenser gueldenstaedtii*) into non-enucleated eggs of the sterlet (*Acipenser ruthenus*), two species bearing different ploidy (4n and 2n, respectively). Up to 12% of the transplanted eggs underwent early development, and one feeding larva (0.5%) was successfully produced. Interestingly, although this transplant displayed tetraploidism (4n) as the donor species, the microsatellite and species-specific analysis showed recipient-exclusive homozygosis without any donor markers. Namely, with regards to this viable larva, host genome duplication occurred twice to form tetraploidism during its early development, probably due to iSCNT manipulation. The importance of this first attempt is to apply iSCNT in sturgeon species, establishing the crucial first steps by adjusting the cloning-methodology in sturgeon’s biology. Future improvements in sturgeon’s cloning are necessary for providing with great hope in sturgeon’s reproduction.

## Introduction

The development of reproductive biotechnology is opening a new window for the conservation of threatened wildlife, as a back up when all other protection policies have failed. In this sense, nuclear transfer, also called cloning, is expected to be a useful tool to preserve species that are nearly extinct or to reconstruct extinct species^1–6^. Interspecific somatic cell nuclear transfer (iSCNT) application to endangered fish species has a great advantage, as the reconstruction of the critically threatened species can be achieved after a single fin-cell is transplanted in the egg-cytoplasmic environment of species whose eggs are easily available in farms. Interspecific cloning using somatic cells has been reported in vulnerable or threatened mammal species like gaur calf, *Bos gaurus*^3,7^, European mouflon, *Ovis orientalis*^5^, black footed cat, *Felis nigripes*^8^ and more recently in Bactrian camel, *Camelus bactrianus*^9^. However, as far as we know, this technique has not been applied to an endangered species. Sturgeons are known as a relict group of species and are amongst the most endangered organisms due to habitat degradation, over-harvesting and illegal fishing for their roe^10,11^. Therefore, it is considered one of the most suitable candidates for cloning in practice. The Acipenseridae family contains species with an interesting specificity; i.e. different ploidy between them. For instance, sterlet, *Acipenser ruthenus*, beluga, *Huso huso* and the European sea sturgeon, *A. sturio* are diploid^12–14^ while the Sakhalin, *A. mikadoi* and Russian sturgeon, *A. gueldenstaedtii* are tetraploid^15,16^ and the shortnose sturgeon, *A. brevirostrum* is hexaploid^17^.

The principle of somatic cell nuclear transfer (SCNT) is that the differentiated donor cell could be restored to full totipotency when it is transplanted in the prosperous environment of a recipient egg^18^. However, the cloning success is limited in fish, even in model species like zebrafish, *Danio rerio*. Since the first adult cloned zebrafish, obtained by Lee, *et al*.^19^ from long-term cultured fibroblast cells, the success of 2% has not been increased using somatic cells. In general, fish SCNT is mostly unsuccessful in producing living adult clones^20–24^. Furthermore, teleost iSCNT yields only poor early embryonic development. This is the case of cloning gynogenetic bighead carp, *Aristichthys nobilis* using gibel carp, *Carassius auratus gibelio* activated eggs that resulted in a success of 27% until blastula stage^25^.

In order to perform the cloning technique, some basic steps must first be established. For instance, optimization of donor cell preparation is one crucial step because cell viability is the primary factor for a successful SCNT. Thus, a piece of fin possesses a high regenerative capacity^26^ and it is easily available, causing minimum damage to the fish^6,26^. This is especially valuable in the case of critically endangered species like sturgeon. Furthermore, the fin-tissue can be harvested even before sexual maturation of the individual^27^ and this is very important for sturgeon species like the beluga whose first maturation usually comes at ~20 years. Another important question for SCNT is whether an extender solution is needed for both egg washing and the working medium. In sturgeon, the eggs need to be washed prior to micromanipulation as they come with coelomic fluid of high viscosity that contains a lot of ovarian somatic and blood cells. To prevent the possibility of transplanting an ovarian somatic cell into the recipient egg, the eggs need to be washed with a physiological saline solution first. During the SCNT procedure, it is convenient that both donor cells and recipient eggs be placed in the same petri dish with the extender solution. Therefore, the medium should not be toxic to any of them, and it must not trigger or enhance egg activation^28^. Just after SCNT, the nuclear transplants (NTs) need to be incubated for a certain period of time before the activation is triggered, so that the donor nucleus can be given the opportunity to equilibrate in the new environment and be reprogrammed^27^.

For sturgeon cloning, to use non-enucleated eggs seems to be preferable, because an enucleation of sturgeon eggs can be harmful and requires time and complicated procedures. In addition, putative spontaneous egg-enucleation^22^ could result in the normal development of the reconstructed embryo^29^. According to Le Bail, *et al*.^28^, transplantation via the micropyle without egg activation seems to improve the efficiency of cloned fish production. However, in this case, a microneedle must be customized for the egg chorion penetration. Finding the correct way of inserting the microneedle, and at which depth within the egg, is a real challenge due to the big size of sturgeon eggs: e.g. sterlet egg, 1.8–2.8 mm; Russian sturgeon egg, 2.8–3.8 mm^30^. Nevertheless, the microinjection position is highly important, as the donor fin-nucleus must be at a favourable place in the host environment in order to be reprogrammed and to produce a cloned sturgeon.

Within the Acipenseridae family, the Russian sturgeon that is classified as critically endangered^11^ is the most preferred for caviar consumption^30^. Despite the advantage of a large amount of eggs per kg of body-weight (10,000–15,000), the drawback of the species is the late age at which the first gonad maturation is settled, 10–16 and 8–13 for females and males, respectively^30^. As a recipient species, the sterlet is promising. Indeed, although it is classified as a vulnerable species^11^, it is considered to be a model species for Acipenseridae as it is one of the smaller sturgeon species and is easily assessable in fish farms. The most important benefit is that its sexual maturity is reached earlier than other sturgeon species, at age 5–8 and 3–5, for female and male, respectively. Additionally, spawning takes place in cycles of 1–2 years in both sexes, which is much more often than other species within the same family^30^.

Overall, the SCNT methodology is a very delicate procedure that requires optimization of many experimental conditions with precise techniques and skilful manipulations. Therefore, the aim of this study was to test the possibility whether the iSCNT can be applied to real endangered species. For this purpose, individuals from Russian sturgeon were used as somatic cell donor species, whereas individuals from sterlet were used as recipient egg providers. The crucial steps of SCNT were examined with these sturgeon species in practice, using shallow-injection of Russian sturgeon’s single fin-cells into the animal pole of sterlet’s non-enucleated and non-activated eggs.

## Results

### Extender solution

**Figure 1:**
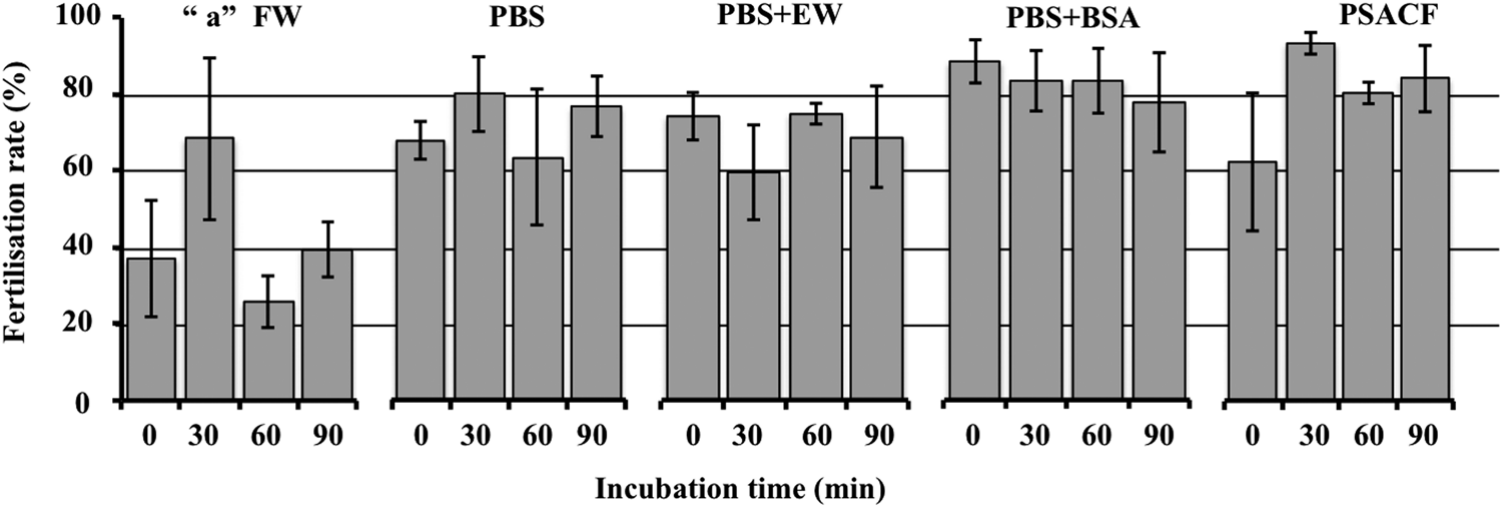
Comparison of extender solutions for egg preservation. Five solutions: filtrated water (FW), phosphate buffered saline (PBS), PBS with egg-white (EW), PBS with bovine serum albumin (BSA) and Persian sturgeon artificial coelomic fluid (PSACF) were tested for their ability to maintain the eggs inactivated. Eggs were washed and incubated for different duration (0 min, 30 min, 60 min or 90 min) in their respective solution. Each value represents the mean ± s.d. fertilization rate (%) from 3 different spawns. Letter “a”: Significant difference (p < 0.001) of control (FW) is indicated by Tukey’s HSD.

Four extender solutions and filtrated water (FW) were tested to find the efficient solution that is able to maintain the recipient eggs inactivated (meiosis II stage) during micromanipulation. All four extender solutions tested gave fairly high fertilization rates (Fig. 1). Whatever the extender solution, the incubation time did not significantly alter the egg ability to be fertilized afterwards. Significant differences (F = 13.660, p < 0.001) in fertilization rate were detected among extender solutions. All extender solutions showed higher fertilization rates compared to the FW group (Tukey’s HSD p < 0.01) but no statistically significant differences were detected among them (Tukey’s HSD p > 0.05). It indicates that some activation occurred when the eggs were manipulated in this medium, and that it affected their ability to be fertilized. From these results, and in order to standardize our working solution, we chose to use Persian sturgeon artificial coelomic fluid (PSACF) and 30 min of incubation for sturgeon-SCNT. Thus, it showed the highest fertilization rates and less variable results after 30 min of incubation (Fig. 1), which is the duration of the SCNT micromanipulation procedure. In fact, there was no sign of egg-activation (no elevation of chorion in sturgeon eggs) while they were kept in PSACF during the whole SCNT procedure.

### Somatic cell nuclear transfer

**Figure 2:**
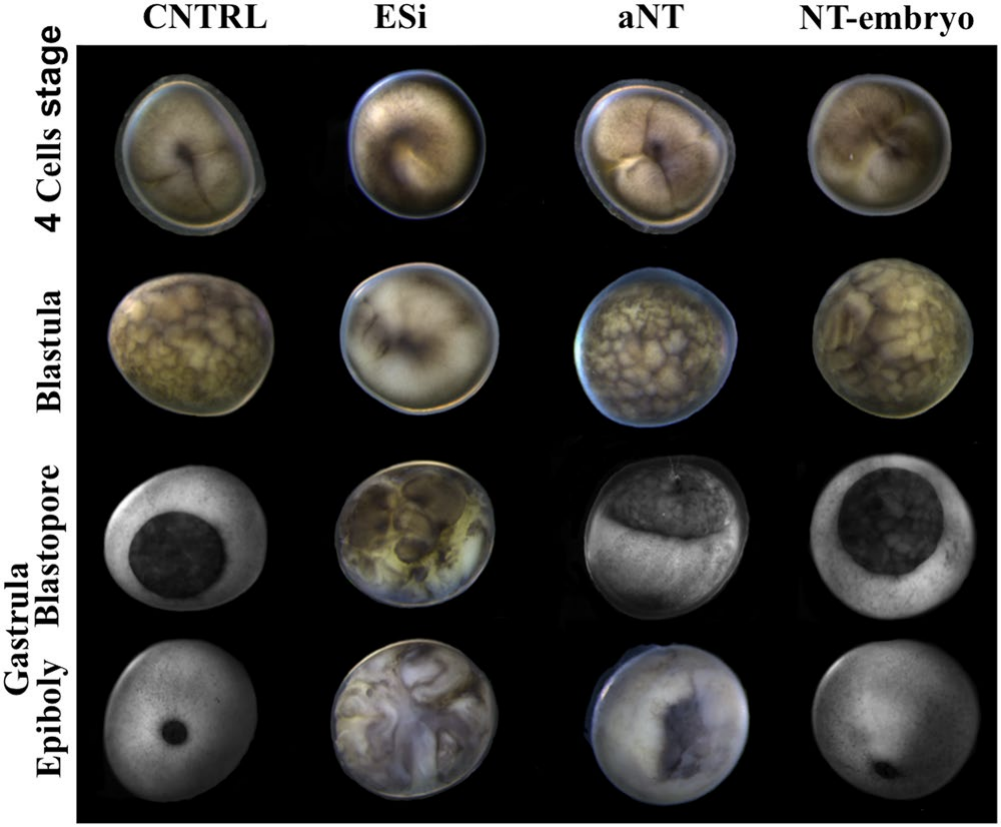
After iSCNT, comparison of early development between a fertilized sterlet embryo from the control group (CNTRL), an extender injected sterlet egg (ESi), an NT-embryo that stopped development at a later stage (2/3 epiboly in gastrula stage)-arrested NT (aNT) and the NT-embryo that surpassed the gastrula stage (completion of epiboly). The aNT-embryo exhibited normal cleavage up to gastrula stage and formation of the blastopore but didn’t reach the epiboly. The NT-embryo exhibited normal cleavages, blastopore formation, and the completion of the gastrulation with a similar pattern to the CNTRL.

**Figure 3:**
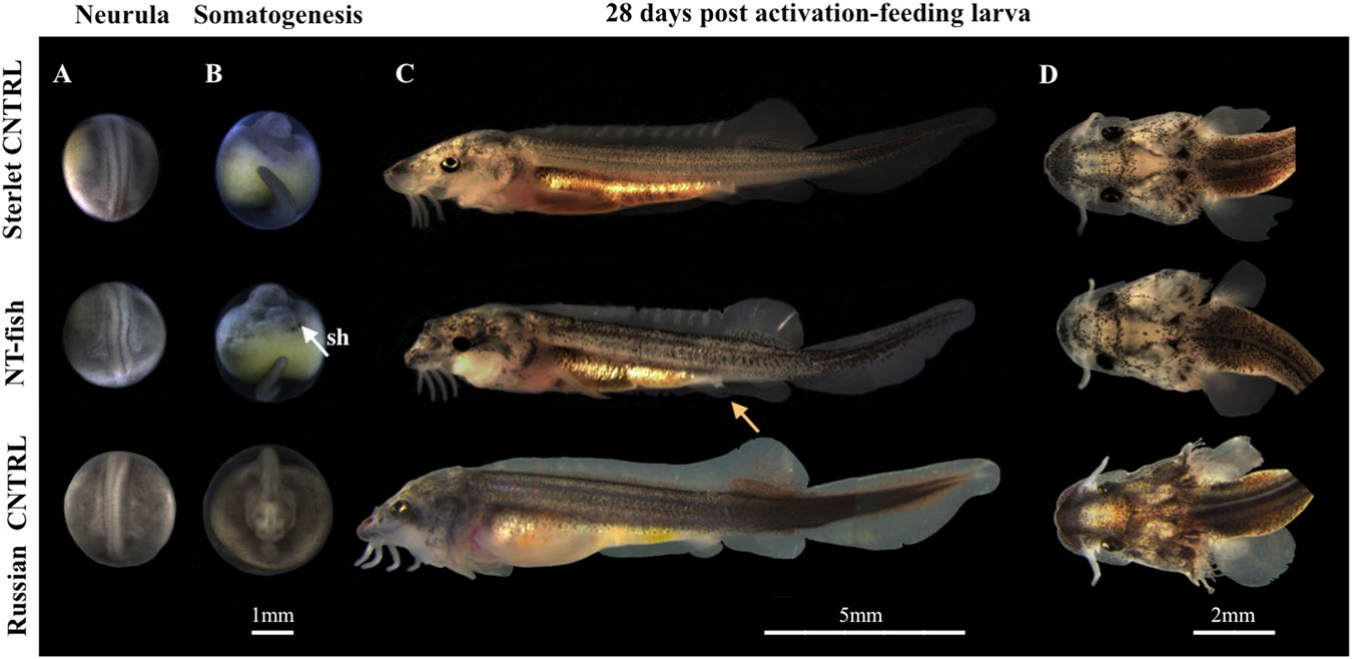
Comparison of the development of sterlet CNTRL, NT-fish obtained after iSCNT and Russian CNTRL from the neurula stage. (**A**) At neurula stage the NT-embryo showed a comparatively normal embryonic development, although its shape seemed to be slightly distorted compared to that of CNTRLs, as the neural fold is bent. (**B**) In the somatogenesis period, the heart of NT-fish showed swelled shape (swelled heart, sh), compared to sterlet and Russian CNTRLs. (**C**) The sterlet and Russian CNTRLs, and the NT-larva at 28 days post activation. At this stage, the slightly distorted body of NT fish observed at neurula stage becomes inconspicuous, providing normal swimming performance. The swelled heart observed in somatogenesis stage in the NT-fish becomes as normal as seen in Russian and sterlet CNTRLs. The stomach of the NT-larva is filled with food (orange arrow). (**D**) The pigmentation pattern of NT-fish is similar to that of sterlet CNTRL.

After the dissociation of fin-tissue from albino sterlet or Russian sturgeon by trypsinization, the cell density was 270,000 ± 8,000 or 275,000 ± 10,000 cells/mL, respectively, with an average cell viability of 95 ± 5% (5 replicates each). Using albino sterlet fin-cells, 129 sterlet eggs reconstructed, from which 13 NTs (10.1%) exhibited initial cleavages and reached the blastula stage. Only 4 embryos (3.1%) reached the gastrula stage and stopped development (Table 1). Using Russian sturgeon fin-cells, in total 210 sterlet eggs submitted to iSCNT and 14 NTs (6.7%) showed sequential cleavages with a normal pattern up to the blastula stage. Among them, some embryos stopped their development and only 10 NTs (4.8%) reached the gastrula stage, and exhibited the blastopore on the margin between the animal and the vegetal hemisphere (Fig. 2; see aNT). While 7 embryos stopped their development and blastomeres were broken down on the course of gastrulation, 3 (1.4%) completed this step and reached the neurula stage. Finally, 1 NT-embryo (0.5%) successfully formed the neural fold and somites (Fig. 3A) and entered the somatogenesis stage that displayed swelled-shape heart (Fig. 3B; see Supplementary Videos S1 and S2). The NT successfully hatched out and fed (Table 1; Fig. 3C; see Supplementary Video S3) while its pigmentation pattern was similar to that of the sterlet control (CNTRL) (Fig. 3D). After three times washing with PSACF and 30 min incubation, more than 50% CNTRL embryos fertilized (n = 251) and developed normally and reached the feeding stage (n = 230) (Table 1). All extender solution-injected CNTRLs (n = 50) did not exhibit development as no somatic cell was injected (Table 1; Fig. 2; see ESi).

**Table 1:** Developing rates of reconstructed embryos after albino sterlet fin-cells transfer (Albino-NTs) or Russian sturgeon fin-cells transfer (Russian-NTs) into sterlet eggs and CNTRLs at each stage.

Experimental group	Total eggs	Blastula (%)	Gastrula (%)	Neurula (%)	Hatching (%)	Feeding (%)
Albino-NTs	129	13 (10.1)	4 (3.1)	0 (0.0)	0 (0.0)	0 (0.0)
Russian-NTs	210	14 (6.7)	10 (4.8)	3 (1.4)	1 (0.5)	1 (0.5)
Fertilized CNTRL	450	251 (55.8)	243 (54.0)	230 (51.1)	230 (51.1)	230 (51.1)
Extender-injected CNTRL	50	0 (0.0)	0 (0.0)	0 (0.0)	0 (0.0)	0 (0.0)

### Flow cytometry and Genotyping

**Figure 4:**
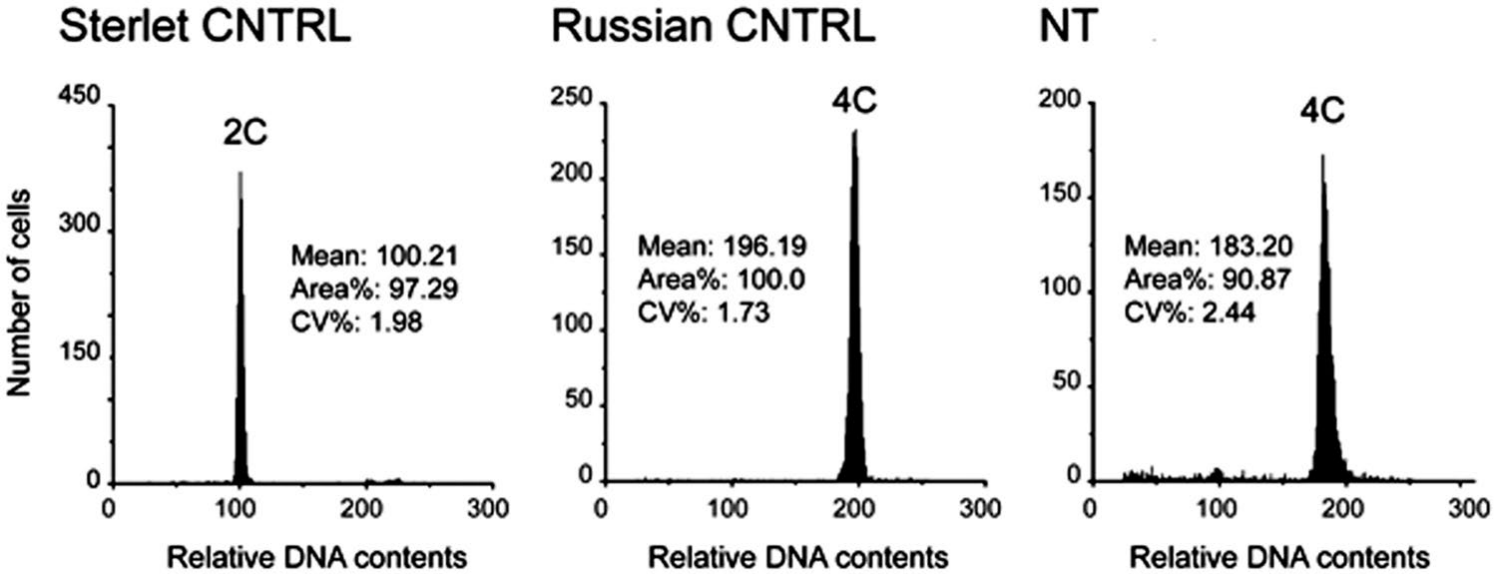
Relative ploidy levels of one sterlet control (CNTRL), one Russian control (CNTRL) and of the 28 days old NT that obtained after iSCNT. Two ploidy levels were observed: 2n in sterlet CNTRL, 4n in Russian CNTRL and 4n in NT-larva. The profiles shown for the CNTRL both sterlet and Russian are similar for all the tested fish.

The ploidy of sterlet CNTRL, Russian CNTRL and of the hatched NT-fish obtained after iSCNT were analysed by flow cytometry. The relative ploidy levels of all sterlet CNTRLs and Russian CNTRLs showed 2n and 4n, respectively (Fig. 4). The cells from the NT-fish at 28 days post activation showed 4n as Russian CNTRL (Fig. 4).

**Figure 5:**
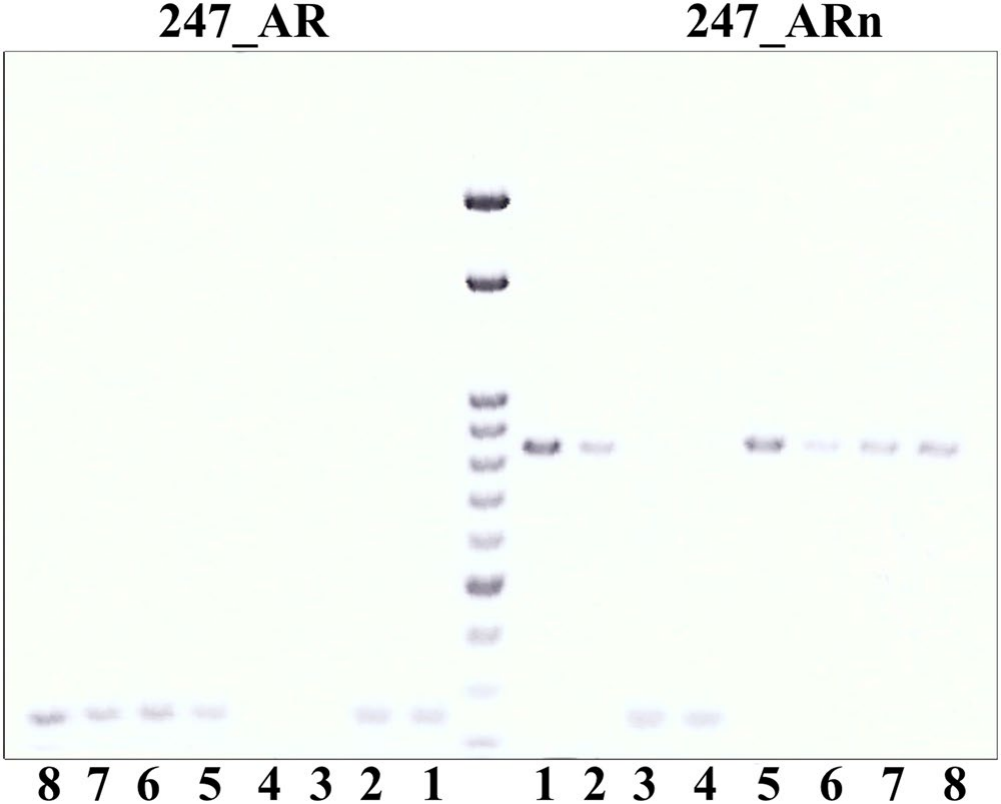
Results of amplification of all samples after iSCNT by sterlet specific primer 247_AR and sterlet negative primer 247_ARn. The most important samples (NT-fish and Russian sturgeon donor) were amplified twice in 2 independent replicates. 1–2 = NT-fish; 3–4 = Russian sturgeon donor; 5 = sterlet recipient; 6 = sterlet male; 7–8 = controls. 750 bp band by 247_ARn primer is not specific and does not affect results.

Using sterlet positive primer pair 247_ARp + 247_uni, we obtained amplification of a 247 bp fragment in NT-fish, sterlet-recipient, sterlet male, and sterlet CNTRL embryos, while no amplification in Russian-donor, as expected (Fig. 5). On the contrary, no amplification in NT-fish, sterlet recipient male, and sterlet CNTRLs, but amplification of a 247 bp fragment in Russian-donor, was observed when using sterlet negative primer pair 247_ARn + 247_uni (Fig. 5). It clearly showed that NT-fish contains only sterlet genome. It was consistent with results of microsatellite genotyping, which confirmed no presence of any allele specific to Russian sturgeon in genotypes of NT-fish (Table 2). More interestingly, NT-fish was fully homozygous at all genotyped loci, including loci where sterlet recipient was heterozygote (Table 2).

**Table 2:** Microsatellite genotyping results.

	Afu_19		Afu_68					Spl_107				
NT-fish	158					232				302		
NT-fish	158					232				302		
Russian-donor		167	148	164	212		248	286		302		318
Russian-donor		167	148	164	212		248	286		302		318
Sterlet-recipient	158					232				302	310	
Sterlet-male	158								298		310	
CNTRL 1	158									302	310	
CNTRL 2	158								298		310	
	**Aox_27**				**Spl_163**			**Spl_173**				
NT-fish		138			214			238				
NT-fish		138			214			238				
Russian-donor	134		154	158		222			240		268	
Russian-donor	134		154	158		222			240		268	
Sterlet-recipient		138			214		226	238		254		
Sterlet-male		138				222		238		254		
CNTRL 1		138				222	226	238		254		
CNTRL 2		138				222	226	238		254		
	**Aox_45**											
NT-fish			156									
NT-fish			156									
Russian-donor	134	137		159	184							
Russian-donor	134	137		159	184							
Sterlet-recipient			156	159								
Sterlet-male			156	159								
CNTRL 1			156	159								
CNTRL 2			156	159								

The NT-fish obtained after iSCNT does not possess any allele specific for Russian-donor genome. NT-fish is homozygous at all loci, including loci, where sterlet female recipient is heterozygote. NT-fish and Russian-donor were analysed in two independent replicates. CNTRL 1 and CNTRL 2 are sterlet control embryos at the same age of NT-fish that obtained after *in vitro* fertilization of Sterlet-recipient and Sterlet-male.

## Discussion

Our experiment is utilizing the interspecific cloning technique in a real endangered species. Using the Russian sturgeon as the donor fin-cell species and the sterlet as the recipient egg species we could establish the basic steps of the nuclear transfer technique. We succeeded to obtain 12% NT early development (i.e. 6.7% NT development in comparison to 56% CNTRL development). Such survival provides great hope for future success in sturgeon-iSCNT.

We decided to work as Siripattarapravat, *et al*.^23^ and Le Bail, *et al*.^28^ on non-activated eggs. Indeed, postponing the egg activation after nucleus injection was shown to increase the NT success when compared to immediate activation^28^. We believe that donor nucleus exposure to the non-activated egg factors is favourable to reprogramming. One reason is that non-activated eggs have high mitosis-promoting factor (MPF) activity^31^ that is likely favourable to donor cell reprogramming. It cannot be excluded either that the resting time that we provided to the clone before triggering meiosis resumption and first mitosis allowed some recovery from the mechanical disturbance induced by the nucleus injection. This put us at some distance from most of the other works done in fish that have used activated recipients^19,25^.

In the present research, we used non-enucleated sterlet eggs according to Wakamatsu,^22^ and Le Bail, *et al*.^28^. It has been suggested that there is an unknown mechanism that allows the female nuclear DNA to be lost after nuclear transfer in medaka, *Oryzias latipes*^32^ or in goldfish, *Carassius auratus*^27^. Indeed, Gasaryan, *et al*.^33^ observed high percentage of diploids from donor origin in the non-enucleated transplants (up to 70%) in loach, *Misgurnus fossilis*. One advantage of skipping the enucleation step is that almost twice as many eggs can be treated in the same experiment^27^. When comparing the use of enucleated and non-enucleated eggs from goldfish and bitterling, *Rhodeus sinensis*, no significant difference has been shown in the development of NTs embryos and hatchlings after transplantation of donor embryonic cell^34^. Also, no significant difference has been observed in early development of NTs after transplantation of somatic cells from gynogenetic bighead carp into enucleated or non-enucleated gibel carp eggs^25^. In the study of Liu, *et al*.^25^, the non-irradiated group exhibited the same development (27.27%) as the treated group (25.71%) at the blastula stage. In our work, we preferred to avoid enucleation because of the risk of damage or losses of the maternal materials (i.e. proteins, mRNAs, mitochondria) that are necessary to support the developing embryo. Since SCNT is a complicate procedure and the developmental rates of the resulted transplants are at stake, we found it preferable to use non-enucleated eggs because they have the same or better result than eggs after mechanical enucleation.

The first and perhaps the most crucial step for a successful SCNT is the use of a suitable extender solution that will maintain the ovulated eggs in metaphase II stage, and that will not be damaging to the fin cells during the micromanipulation. In some fish species, coelomic fluid is used for SCNT. For instance, salmonid ovarian fluid has proved to be suitable to maintain zebrafish^31,35,36^ and goldfish^37^ eggs in an inactivated state. Our results demonstrated that sturgeon eggs can remain inactivated even though they are incubated into a saline solution. This is quite advantageous in our species, because sturgeon coelomic fluid quality varies among females. Additionally, besides the blood vessels and follicular cells that it contains, its density is so high that it prevents the visualization of the donor cell and its aspiration through the microneedle. Therefore, sturgeon coelomic fluid is not a good candidate to be used in iSCNT. In order to standardize our cloning technique, we chose to use PSACF because our results demonstrated that the egg ability to be fertilized afterwards remained high during the experimental timeline. We also chose to incubate our transplants for 30 min after nuclear transfer before activation, because it has been reported that incubation of eggs after transplantation for a certain period improves the developing rate of clones, probably by prompting the reprogramming of the donor nuclei^23,28^. According to Le Bail, *et al*.^28^, hatching stages of goldfish clones were reached only when the nucleus was incubated for at least 30 min prior to egg activation. In sturgeon-iSCNT, such a 30 min- incubation is favourable as this time facilitates the micromanipulation of such a large egg (~20 nuclear transfers).

It was striking to observe that all sturgeon NT-embryos that showed initial cleavage furrows continued development through the mid-blastula stage, and that many of them developed up to the gastrula stage. This result is in contrast with previous studies showing that a high percentage of the NT face difficulties to overcome the mid-blastula stage^22–24,28,38^. The later has been reported to be the stage at which the mitotic checkpoints are established^39^, and at which the embryonic genome is activated^40^. Failure to develop further than the mid-blastula stage is inferred to the incomplete epigenetic resetting of the donor nucleus in the egg environment that results in inaccurate zygotic genes expression^24^. Before this stage, embryos develop with maternally supplied factors^41,42^. We noticed that in sturgeon-SCNT, the critical embryonic stage of the NTs seems to be the completion of gastrula. However, we cannot give an efficient explanation for this result since we lack information concerning the stage at which the embryonic genome is activated in our species and when it could affect the developing embryo.

From our results and from previous studies in fish, the SCNT technique doesn’t give very hopeful results. The percentage to obtain clone animals even in model fish species varies and is generally low as it is for sturgeon. Cloning success in obtaining embryos after non-enucleation of goldfish egg is 17%^28^, after enucleation of zebrafish egg the cloning success in obtaining adult individuals is 2%^19^. In addition, cloning success in medaka after transplantation of fresh harvested fin-cells into diploidized eggs gave 2.7%^43^. Liu, *et al*.^25^ discussed that crossing species with different ploidy is an obstacle for embryonic development and this could be a reason of Russian-NT’s low success in our study. Even though we are aware that hybridization in Acipenseridae family is high, we do not have information about fertilization rate using sterlet egg (1n) and Russian sturgeon sperm (2n), because the studies on sturgeon are using combination of parents with the same ploidy level. In addition, we still lack sufficient information to explain the difference in the degree of development after iSCNT in sturgeon from the other fish species.

Although we know, from our experience, that the sturgeon embryos display high variability of developmental speed, even in the same batch of eggs that were fertilized simultaneously, we cannot conceal that after iSCNT some NT-embryos and the resulted NT-larva, exhibited slightly faster development than the CNTRL mainly after the blastula stage. These Russian-NTs developed faster than the CNTRL and with a difference of 2 hours. However, the NT-larva developed faster than CNTRL from the first cleavage until the beginning of the neurula stage, which synchronized with the CNTRL’s speed. According to Newport and Kirschner^44^, the quicker development of the NT-larva comparing to CNTRL can be explained by the higher NT’s ploidy level (double than CNTRL). These researchers demonstrated that *Xenopus* embryos of higher ploidy underwent the MBT at correspondingly earlier times than diploid embryos used as controls. However, the faster development of some NT-embryos does not mean that all of them displayed the same DNA pattern of NT-larva. Thus, some NT-embryos may be real clones, exhibiting the ploidy of Russian sturgeon (4n) or even chimeras, exhibiting both genomes due to fusion of Russian somatic cell with the sterlet egg (4n + 1n = 5n).

Embryonic development of sturgeon species is very similar in pattern of cleavage as well as the morphology of embryo. The larvae of most sturgeon species can be unambiguously identified only by appropriate molecular markers^45^. To uncover, whether NT-fish contains genome of Russian sturgeon donor, we accommodated recently developed nuclear DNA markers^46^. The results of molecular genotyping clearly showed that NT-fish contains only sterlet genome. Thus, any contribution of Russian sturgeon donor to NT-fish, including chimerism, can be excluded.

**Figure 6:**
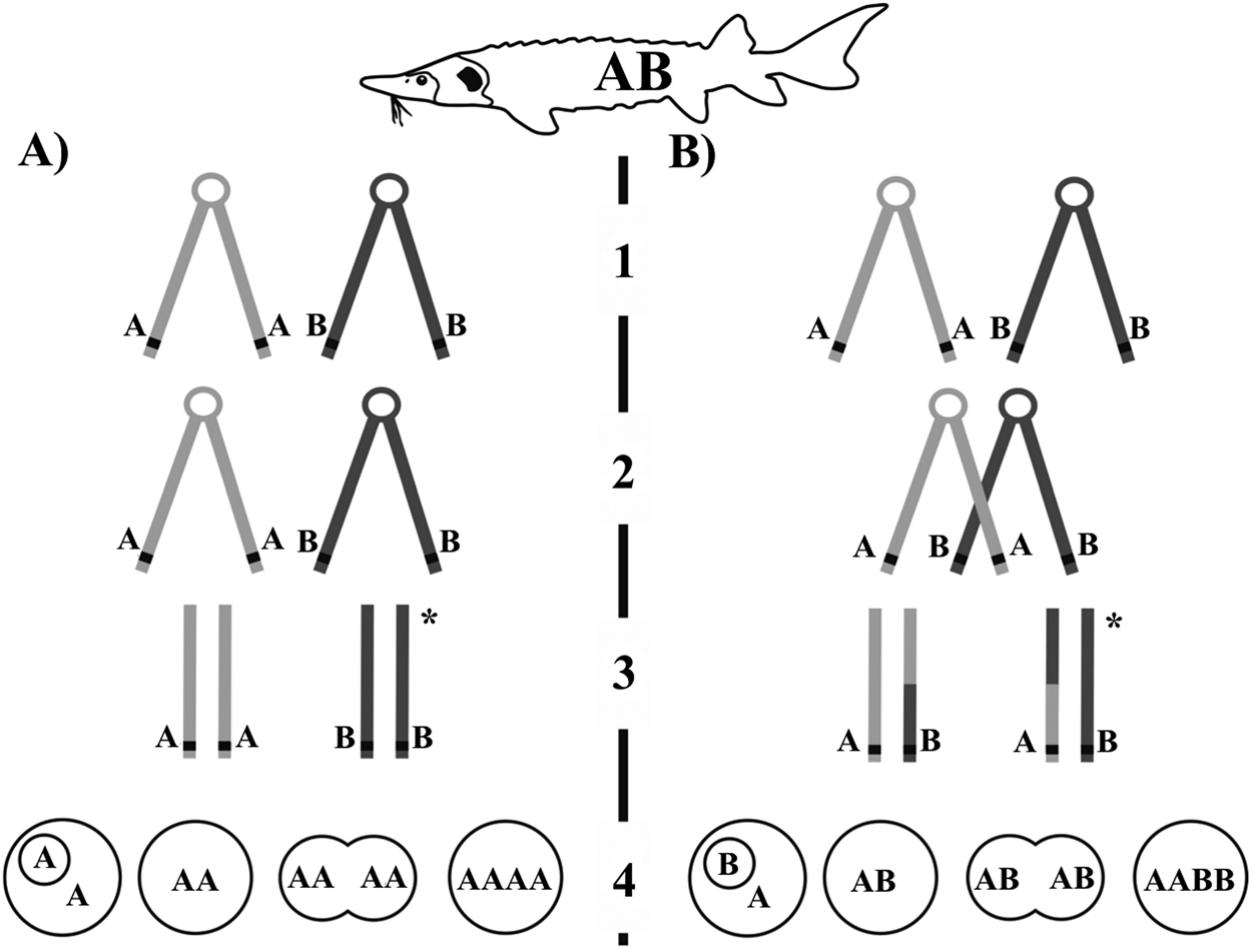
Scheme of meiosis and suggested disturbance in subsequent early embryonic development as possible explanation of NT-fish origin after iSCNT. Sterlet recipient is heterozygote at given locus (AB). (**A**) No crossing over between homologs chromosomes in meiosis followed by retention of 2nd polar body and suppression of first mitotic cleavage results in homozygote individual; (**B**) if crossing over occurs, NT-fish cannot be homozygote under same scenario of post-meiotic development 1 = bivalent of two homologues chromosomes with replicated chromatids; 2 = crossing over between non-sister chromatids occurs in (**B**), while no crossing over in (**A**); 3 = homologues chromosome segregation (anaphase I); *become first polar body. This is followed by standard meiosis II, but second polar body is not released. Thus oocyte is diploid before mitotic cleavage starts; 4 = retention of second polar body and suppression of first mitotic cleavage result in tetraploid homozygote (AAAA) if there is no crossing over in meiosis I - (**A**), but in heterozygote tetraploid (AABB) if crossing over occurs (**B**).

Two hypotheses can explain this lack of donor contribution while the occurrence of tetraploidism. The first is that there would have been retention of the second polar body and successive suppression of first mitotic cleavage. This would mean that the transplant underwent both events that are more often triggered separately in fish for biotechnological purposes: triploidisation by retention of the second polar body, and diploidisation by preventing the first mitosis. The nucleus of a mature sturgeon egg is at metaphase II stage^47^, and any kind of physical or chemical shock applied during meiosis II in fish eggs can prevent the extrusion of the second polar body, while still allowing chromosomal division^48^. Microinjection might work as a physical shock. This scenario, however, is the less probable due to the full homozygosity of NT-fish at analysed loci. NT-fish would be homozygous at loci where sterlet-recipient was heterozygote only if there were no crossovers between the locus and its centromere (Fig. 6A). If the crossing happened, the NT-fish could not be fully homozygote (Fig. 6B).

**Figure 7:**
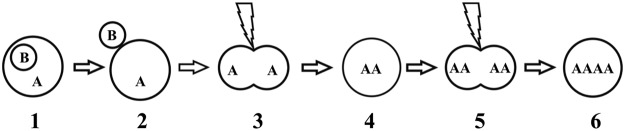
Disturbance in early embryonic development as possible explanation of NT-fish origin after iSCNT. Meiosis occurs normally and second polar body (B) is released after activation of the oocyte (1–2). Whether crossing over occurs or not is not relevant for this scenario because oocyte is haploid before first mitotic cleavage. 3 = suppression of first mitotic cleavage results in diploid homozygote (AA) cell – 4; 5 = suppression of subsequent (second) mitotic cleavage of diploid cell (4) results in tetraploid homozygote (AAAA) cell – 6. This is followed in common embryonic development giving tetraploid, fully homozygote individual.

The second hypothesis, that stands more probable, is the suppression of the first and second mitotic cleavage. Under this scenario, meiosis normally occurred and the second polar body was released after egg activation by injection resulting in the haploid egg. Spontaneous suppression of 1st cleavage resulted in 2n, and another suppression of the following cleavage in 4n cell (Fig. 7). In their striking work on early development in zebrafish, Yabe, *et al*.^49^ showed that when the pair of centrioles brought by the spermatozoa was altered or absent, the first mitosis was inhibited although genome duplication had occurred. This produced a first set of whole genome duplication. These authors also showed that after the first mitosis following normal fertilization, the lack of specific maternal factors involved in centrosome duplication induced transient defects in cellular cleavage, leading to genome doubling in these blastomeres. Interestingly, these blastomeres could be rescued and resume normal cleavage on subsequent mitosis. A parallel situation between this work and our observations on sturgeon cannot be excluded. In our case, the centrosomes brought by the donor cell may have failed to sustain the first mitosis, and duplication of the haploid maternal genome may have then occurred thanks to some sham fertilization signal brought by the SCNT procedure. Next, the medium injected with the donor cell may have transiently diluted the maternal factors necessary for the second cleavage, resulting in a second round of genome duplication without cell division, followed by normal mitosis resumption. The creation of a 4n individual from unfertilized egg of 2n female is a very interesting result and our data are all relevant with the hypothesis that the donor cell-injection provoked a big stress in the egg, causing gynogenesis.

To the best of our knowledge, the present work is the first report of SCNT among sturgeons. The early development that we have observed proves that SCNT is applicable to ancient chondrosteans as it has been applied in modern teleosteans species^21,25^. It is an easy and cheap technique that can be performed by any laboratory with microinjection experts, as far as the sturgeon-eggs and the fin-tissue can be provided. As a side effect of our iSCNT experiments, unusual disruption in early embryogenesis resulted in fully homozygous tetrahaploid, an event that has not been observed in any sturgeon, or teleostean fish or even vertebrate before. This may have unraveled some mechanism involved in polyploidisation in sturgeon species. Even though we did not succeed to produce any true sturgeon clone, we have established several steps that pave the way for future development of iSCNT in sturgeon in the near future. The use of the cytoplasmic environment from an easy-reared fish like the sterlet with small reproductive maturation can regenerate sturgeons with high value for conservation and management like are Russian sturgeon and beluga.

## Methods

### Ethics

The transplantation experiments took place at the Faculty of Fisheries and Protection of Waters, Research Institute of Fish Culture and Hydrobiology, University of South Bohemia in České Budějovice, Czech Republic. All experimental procedures were performed in accordance with national and institutional guidelines on animal experimentation and care. This study was approved by the Animal Research Committee of the University of South Bohemia in České Budějovice. Fish were maintained according to the principles of animal welfare and principles of laboratory animal care based on the Guidelines for the Use of Animals in Research.

### Fish and gamete collection

In order to perform the SCNT technique we used non-sexually matured albino sterlet (2–3 years old) or Russian sturgeon (1–2 years old) as fin-donor species and matured sterlet females (5–6 years old) as egg-recipient species. To perform *in vitro* fertilization of the CNTRL group sperm was collected from matured male sterlet individuals (5–6 years old). Ovulation and spermiation were induced by an intramuscular injection of the gonadotropin-releasing hormone analogue (des-gly10[d-Ala6]-LHRH; Sigma). Eggs and sperm were stripped and fertilized one day after injection. In order to induce ovulation, the sterlet females were injected by a single intramuscular injection of carp pituitary homogenized extract (CPE) at a dose of 5 mg/kg body weight (b.w.) in two steps: first with 0.5 mg/kg b.w. and second with 4.5 mg/kg b.w., 12 h after the first injection. Egg collection was performed 18–20 h after the second injection. In order to induce spermiation, the sterlet males were injected once (4 mg/kg b.w.) with CPE and sperm was collected 48 h after hormonal injection by use of a catheter from the urogenital papilla, transferred to a separate cell culture container (250 mL), and stored at 4°C until sampling (1–3 h).

### Examination of the extender solution suitable for egg manipulation

Five solutions were tested as follows: phosphate buffered saline (PBS; SIGMA-ALDRICH®), PBS + 1% bovine serum albumin (BSA; SIGMA-ALDRICH®), PBS + 1% egg white, PSACF^50^ and FW. According to Sohrabnezhad, *et al*.^50^ PSACF was formulated as 80 mM NaCl, 3.96 mM KCl, 0.78 mM MgSO_4_.7H_2_O, 0.26 mM CaCl_2_, 2.42 mM glucose, 1 g bovine serum albumin, 20 mM NaHCO_3_, 20 mM Hepes buffer (4–2-hydroxy-ethyl-1-piperazineethanesulfonic acid), the osmotic pressure was adjusted to 250 mOsm/L and the final pH of the medium was adjusted to 7.5 using 1 N, NaOH. All solutions were kept at 15 °C during experimentation. Three replicates (eggs from 3 sterlet individuals) were performed for each tested group. For each replicate, 17 to 60 eggs were used for each test. For each tested extender, the eggs were washed three times for five minutes in order to remove debris and somatic cells. Then, eggs were incubated for 0, 30, 60, or 90 min at 15 °C in the tested extenders. *In vitro* fertilization was conducted with sterlet fresh-stripped sperm in order to monitor egg quality after incubation. For fertilization, extender solutions were removed completely, 20 μL sperm were added to each dish, and activated with FW. Fertilization rate was assessed after 8 h (mid-blastula stage) at 15 °C. Differences between extender solutions were analysed using one-way ANOVA test. To determine significant pair-wise differences between treatments Tukey’s HSD post-hoc test was applied.

### Donor fin-cells preparation

A small piece (0.5 cm^2^) of albino sterlet or Russian sturgeon caudal fin was clipped using sterile scissors. The fin fragment was washed with PBS under gentle shaking for one min to remove mucus. One-third of the fin was stored in 96% ethanol for DNA extraction, and the remaining piece was kept for preparation of the donor cells. The fin-tissue was dissociated in 0.5% Trypsin (Gibco® Life Technologies; ThermoFisher SCIENTIFIC) in PBS for 2 h at 15 °C with gentle shaking (TUBE REVOLVER; Thermo SCIENTIFIC). Dissociated cells were filtrated using a 50 µm pore size filter (CellTrics®) and centrifuged at 800xg for 10 min at 15 °C. The cells were then washed twice with PBS containing 0.05% DNase and the last pellet was suspended in 150 µL PBS. Cell viability and concentration were assessed using a haemocytometer (Bright-Line^TM^ Hemacytometer; Hausser Scientific) after Trypan blue staining (SIGMA-ALDRICH®). The cells were kept at 4 °C until use, no longer than 8 h.

### Nuclear transfer

After washing with PSACF three times to remove the debris and somatic cells, eggs were placed in a 6 cm^2^ petri dish filled with the PSACF at 15 °C. The SCNT was conducted by a hydraulic injector (Cell-Tram Oil; Eppendorf, Germany) connected to a micromanipulator (MO-152; Narishige, Japan) under a stereomicroscope (Leica M165 FC.) according to Le Bail, *et al*.^28^. An egg was fixed on a glass capillary holder (inner diameter: 0.7–0.8 mm) so that the animal pole faced the transfer needle, and a small amount (5 μL) of the fin-cell suspension (270 ± 4 or 275 ± 10 cells/μL for albino sterlet or Russian sturgeon, respectively) was dropped near the egg. Then, a single fin-cell was gently aspirated into the hand-made microcapillary (inner diameter: 25–28 μm) and was introduced in the animal pole of the egg, where the micropyles are located. After SCNT using fin-cells from albino sterlet (4 experiments) or Russian sturgeon (6 experiments), the transplants (n = 129 or 210, respectively) were incubated in the PSACF (15 °C) for 30 min and then were activated with FW. Simultaneously, fertilization of the CNTRL group with freshly stripped sterlet sperm was performed to produce CNTRL sterlet embryos (n = 450). For a negative CNTRL, we used eggs injected with the extender solution without a fin-cell, and after 30 min incubation they activated with water (n = 50).

### Cultivation of the embryos

After activation of the nuclear-transplants (NTs) and fertilization of the CNTRL group, all eggs were treated with 0.1% tannic acid (SIGMA-ALDRICH®) for 10 min to remove the surface stickiness. After washing the embryos three times with water, embryos were cultured in FW containing 0.01% penicillin and 0.01% streptomycin for three days at 15 °C. Forceps were used to remove the outer layers of chorion at 10 h post-activation. The inner layer of the chorion was removed at 5–6 days post activation. First feeding started after yolk resorption, 20 days after fertilization. The freshwater annelid worm *tubifex* sp. was given twice per day at 8:00am and 16:00 pm. Tanks were cleaned twice per day 2 h after the meal.

### Ploidy assessment

Fragments of caudal fins from five adult sterlet and two Russian sturgeons, and from the 28-days-old feeding larvae (CNTRL and NT-fish) were used for ploidy assessment. The tissues were minced in extraction buffer and then stained with 4-6-diamino-2 phenylindole dihydrochloride (CyStain DNA 2step kit; Partec GmbH). Ploidy of these samples was determined by flow cytometry (Ploidy Analyser; Partec).

### Molecular genotyping

Fin clips from both cell-donor (Russian sturgeon) and egg-recipient females (sterlet) were collected and fixed, as mentioned above. Genomic DNA was extracted using a DNA extraction kit according to manufacturer’s instructions (GenElute Mammalian Genomic DNA Miniprep Kit; SIGMA-ALDRICH®). Six samples, DNA from NT-fish, sterlet-recipient, sterlet-male, Russian-donor and 2 sterlet CNTRL embryos were analysed.

Samples were tested by sterlet positive primer pair 247_AR + 247_uni and subsequently by sterlet negative primer pair 247_ARn + 247_uni as described in Havelka, *et al*.^46^. If a sample contains only sterlet DNA, the 247 bp fragment is amplified only by sterlet positive primer pair. If a sample contains DNA of other sturgeon species, the 247 bp fragment is amplified only by sterlet negative primer pair. Finally, if a sample contains DNA of sterlet and other sturgeon species (e.g. sample is hybrid or chimera) 247 bp fragment is amplified by both primer pairs in two independent reactions. Briefly, PCR reactions were performed in a total volume of 25 μL containing 0.25 μM of each primer, 75 mM Tris-HCl, pH 8.8, 20 mM (NH_4_)_2_SO_4_, 0.01% Tween 20, 2.5 mM MgCl_2_, 800 μM dNTP, 2.5U Taq-Purple DNA polymerase, and 25 ng of DNA template under following cycling conditions: 95 °C for 120 s; 5 cycles at 95 °C for 60 s, 63 °C for 60 s, and 72 °C for 60 s; 25 cycles at 95 °C for 30 s, 63 °C for 30 s, and 72 °C for 60 s; and a final extension at 72 °C for 12 min. PCR products were inspected on 1.5% agarose gel.

Basic like-parentage assignment was performed using seven microsatellite markers developed for sturgeon species including Afu_19, Afu_68^51^; Aox_27, Aox_45^52^; Spl_107, Spl_163 and Spl_173^53^. Microsatellites were amplified according to protocol of Havelka, *et al*.^54^. Forward primers within each of the 7 primer sets possessed a 5′prime end tail (M13R). During PCR, a fluorescently labelled primer (M13R) was added to the standard amplification reaction. Touchdown PCR protocol was employed for thermal cycling: initial denaturation 95 °C for 3 min followed by 10 cycles of 95 °C for 45 s, 65 °C–1 °C per cycle for 45 s and 72 °C for 45 s, followed by 20 cycles of 95 °C for 45 s, 53 °C for 45 s and 72 °C for 45 s, and final extension 72 °C for 10 min. Microsatellite fragment analysis was performed on a 3130xl ABI Genetic Analyser (Applied Biosystems, TM) using a GeneScan LIZ 500 size standard (Applied Biosystems, TM), and genotypes were scored in GENEIOUS 6.1.8^55^ using Microsatellite Plugin 1.4.

## Electronic supplementary material

Supplementary information

Supplementary Video S1

Supplementary Video S2

Supplementary Video S3
