# Author Correction: Application of interspecific Somatic Cell Nuclear Transfer (iSCNT) in sturgeons and an unexpectedly produced gynogenetic sterlet with homozygous quadruple haploid

**DOI:** 10.1038/s41598-020-58285-z

**Published:** 2020-02-05

**Authors:** Effrosyni Fatira, Miloš Havelka, Catherine Labbé, Alexandra Depincé, Viktoriia Iegorova, Martin Pšenička, Taiju Saito

**Affiliations:** 1 Faculty of Fisheries and Protection of Waters, South Bohemian Research Center of Aquaculture and Biodiversity of Hydrocenoses, University of South Bohemia in Ceske Budejovice, Zátiší 728/II, 389 25, Vodňany, Czech Republic; 2 Faculty and Graduate School of Fisheries Sciences, Hokkaido University, 3-1-1 Minato, Hakodate, Hokkaido, 041-8611 Japan; 3 INRA, Fish Physiology and Genomics department, Campus de Beaulieu, F-35000 Rennes, France; 4 Nishiura Station, South Ehime Fisheries Research Center, Ehime University, Uchidomari, Ainan, Ehime, 798-4206 Japan

Correction to: *Scientific Reports* 10.1038/s41598-018-24376-1, published online 16 April 2018

The original version of this Article contained a typographical error in the Abstract.

“Up to 6.7% of the transplanted eggs underwent early development, and one feeding larva (0.5%) was successfully produced.”

now reads:

“Up to 12% of the transplanted eggs underwent early development, and one feeding larva (0.5%) was successfully produced.”

In addition, the Acknowledgement section contained a typographical error.

“The study was financially supported by the Ministry of Education, Youth, and Sports of the Czech Republic; projects “CENAKVA” (South Bohemian Research Center of Aquaculture and Biodiversity of Hydrocenoses; CZ.1.05/2.1.00/01.0024) and “CENAKVA II” (LO1205 under the NPU I program); and the Czech Science Foundation (P502/13/26952 S). The study was also supported by the Grant Agency of the University of South Bohemia in České Budějovice (GAJU Fatira 068/2017/Z) and was partially funded by the French CRB Animal project ≪Investissements d’Avenir≫, ANR-11-INBS-0003. The support of the EU COST Actions FA1205: AQUAGAMETE is gratefully acknowledged. We are in debt to Ian AE Butts for the English language editing and Martin Flajšhans for his scientific inputs.”

now reads:

“The study was financially supported by the Ministry of Education, Youth, and Sports of the Czech Republic; projects “CENAKVA” (South Bohemian Research Center of Aquaculture and Biodiversity of Hydrocenoses; CZ.1.05/2.1.00/01.0024) and “CENAKVA II” (LO1205 under the NPU I program); and the Czech Science Foundation (17-19714Y). The study was also supported by the Grant Agency of the University of South Bohemia in České Budějovice (GAJU Fatira 068/2017/Z) and was partially funded by the French CRB Animal project (Investissements d’Aveni), ANR-11-INBS-0003. The support of the EU COST Actions FA1205: AQUAGAMETE is gratefully acknowledged. We are in debt to Ian AE Butts for the English language editing and Martin Flajšhans for his scientific inputs.”

These errors have now been corrected in the PDF and HTML versions of the Article.

