# Supplementary material for: Application of interspecific Somatic Cell Nuclear Transfer (iSCNT) in sturgeons and an unexpectedly produced gynogenetic sterlet with homozygous quadruple haploid
Source: Sci Rep. 2018 Apr 16;8:5997. doi: 10.1038/s41598-018-24376-1 (PMC5902484; doi:10.1038/s41598-018-24376-1)
Supplement: Supplementary file 1 — Supplementary information [file 41598_2018_24376_MOESM1_ESM.pdf]

Supplementary Information for manuscript SREP-17-45106A

Application of interspecific Somatic Cell Nuclear Transfer (iSCNT) in sturgeons and an unexpectedly produced gynogenetic sterlet with homozygous quadruple haploid

Effrosyni Fatira[1,*], Miloš Havelka[1,2], Catherine Labbé[3], Alexandra Depincé[3], Viktoriia Iegorova[1], Martin Pšenička[1], Taiju Saito[1,4]

[1]Faculty of Fisheries and Protection of Waters, South Bohemian Research Center of Aquaculture and Biodiversity of Hydrocenoses, University of South Bohemia in Ceske Budejovice, Zátiší 728/II, 389 25 Vodňany, Czech Republic
[2]Faculty and Graduate School of Fisheries Sciences, Hokkaido University, 3-1-1 Minato, Hakodate, Hokkaido 041-8611, Japan.
[3]INRA, Fish Physiology and Genomics department, Campus de Beaulieu, F-35000 Rennes, France.
[4]Nishiura Station, South Ehime Fisheries Research Center, Ehime University, Uchidomari, Ainan, Ehime 798-4206, Japan.

*Corresponding author: effrosini.fatira@gmail.com
Tel.: +420 38777 4785

Video S1. Frontal view of NT-fish. Swelled heart is beeping at 5 days post activation.

Video S2. Lateral view of NT-fish. Swelled heart is beeping at 5 days post activation.

Video S3. 27 days post activation. The NT-larva is being fed with *tubifex* sp.
